# Study on the correlation between dietary patterns and bone health among rural elderly people in Qingdao

**DOI:** 10.3389/fnut.2025.1613065

**Published:** 2025-09-05

**Authors:** Haoran Chang, Wenchao Han, Jiafei Li, Dazhi Jiao, Fangyu Liu, Tianlin Gao, Wenjing Zhu, Jianbao Gong

**Affiliations:** ^1^School of Public Health, Qingdao University, Qingdao, China; ^2^Department of Pediatrics, Qingdao Hospital, University of Health and Rehabilitation Sciences (Qingdao Municipal Hospital), Qingdao, China; ^3^Department of Medical Research, Qingdao Hospital, University of Health and Rehabilitation Sciences (Qingdao Municipal Hospital), Qingdao, China; ^4^Department of Orthopedics Surgery, Qingdao Hospital, University of Health and Rehabilitation Sciences (Qingdao Municipal Hospital), Qingdao, China

**Keywords:** elderly, bone mineral density, composition of body, osteoporosis, dietary pattern

## Abstract

**Objective:**

To evaluate the relationship between dietary patterns and bone health in the elderly, and to guide the elderly to establish a reasonable dietary pattern and improve bone health.

**Methods:**

This cross-sectional study included 544 elderly individuals aged 60 and above in rural areas of Qingdao. Dietary patterns were analyzed using factor analysis. A logistic regression model was employed to assess the relationship between dietary patterns and osteoporosis. The correlation between body composition and T-score was analyzed using Spearman's rank correlation method in the R software.

**Results:**

A total of four main dietary patterns were identified in this study, which included a balanced dietary pattern, a high-protein dietary pattern, a condiment dietary pattern, and a snack dietary pattern. After adjusting for potential confounding factors (age, residence status), it was found that a high-protein dietary pattern was positively correlated with bone health at the Q3 level (OR (95%CI) 0.435(0.190, 0.997), but not correlated at the Q4 level. The high-protein dietary pattern was significantly positively correlated with several body composition parameters, including intracellular fluid, extracellular fluid, total body water, protein, muscle mass, minerals, fat-free mass, skeletal muscle, left-hand muscle mass, right-hand muscle mass, and trunk muscle mass (all *P* < 0.05). Additionally, these body composition factors were positively correlated with osteoporosis T-scores.

**Conclusion:**

There is a correlation between dietary patterns and osteoporosis; a moderate amount of the high-protein dietary pattern is beneficial to the bone health of the elderly.

## 1 Introduction

Osteoporosis is a systemic bone disease influenced by both bone mass in early adulthood and the subsequent rate of bone loss (1). Bone mineral density (BMD) is a key determinant of osteoporosis (2). A 10% increase in peak bone mass (PBM) has been reported to reduce the risk of fracture later in life by 50% (3). Individuals in middle age and the elderly face a heightened risk of osteoporosis, given the accelerated bone loss associated with aging (1). In a 2021 study, P-L Xiao et al. conducted a comprehensive review of the global prevalence of osteoporosis. The findings revealed a 30.5% prevalence of osteoporosis in individuals aged 50 and above worldwide (4). Notably, in China, the prevalence of osteoporosis among those aged 60 to 69 was reported to be 35.2%, while among those aged 80 and above, the prevalence rate of osteoporosis is as high as 53.9% (5). Therefore, osteoporosis in the elderly population in China warrants serious attention to prevent its progression to bone damage, underscoring the urgency of addressing this issue to prevent bone damage and alleviate associated medical burdens on families and society.

BMD and the development and progression of osteoporosis are influenced by Genetics, endocrinology, machinery, lifestyle, and more (6–10). Among various lifestyle factors, diet is considered the most modifiable risk factor (11). Hence, the elderly population needs to take intervention measures about increasing or preventing a decrease in BMD to prevent osteoporosis. For instance, calcium and vitamin D, in the form of calcium phosphate, play a vital role in the bone mineral matrix, contributing significantly to bone strength (12). Dairy products, being rich in calcium and protein, have the potential to lower parathyroid hormone (PTH) levels, thereby positively impacting bone remodeling (13–17). Despite the established beneficial effects of specific nutrients and foods on bone health, some studies overlook the intricate relationships and interactions among different dietary components. People eat meals consisting of a variety of foods with complex combinations of nutrients (18). And some nutrients (such as potassium and magnesium) are highly correlated, making it difficult to examine their separate effects (19). In addition, the effect of a single nutrient may not be detected because its effect is too small (20). Therefore, it is essential to explore the potential connections between dietary patterns and bone health.

Since 2002, research on the association between dietary patterns and skeletal health has been a focal point in public health (21–25). Osteoporosis is a major clinical problem in elderly people (26), yet despite growing recognition of dietary patterns' importance for their bone health, few studies have used principal component analysis to examine this association in elderly populations across diverse regions, with inconsistent results (27–30). Among these, the impact of the Mediterranean diet on bone density (BMD) has garnered significant attention. The Mediterranean diet is characterized by the extensive consumption of unrefined grains, fruits, vegetables, beans, and olive oil (9). Most studies have shown that adherence to MD was associated with a higher BMD (31–33). While some cohort studies suggest that the Mediterranean diet may have no effect or even adverse effects on bone health (34–37), further research on dietary patterns is necessary.

In our study, we conducted a cross-sectional investigation in Qingdao, China, using principal component analysis to explore the correlation between dietary patterns and bone mineral density (BMD), along with body composition. Through this study, we aim to identify specific dietary patterns that could help mitigate the risk of osteoporosis in the elderly population of Qingdao, China, providing a foundation for targeted dietary interventions in osteoporosis prevention.

## 2 Subjects and methods

### 2.1 Subjects

A cross-sectional study was conducted in the town of Jiaozhou Riku from April to July 2019, encompassing a random sample from 18 predominantly farmer villages. The research team recruited 806 participants through face-to-face interviews. All research staff received standardized training to ensure consistent data collection procedures. The inclusion criteria comprised individuals who were (1) aged 60 years or older, (2) with a history of local residence of at least 10 years, and (3) willing to participate in the study. Exclusion criteria were defined as follows: (1) individuals with conditions affecting cognitive function (e.g., alcoholism, stroke, and cerebral infarction), (2) individuals with neurological diseases (e.g., Alzheimer's disease, Parkinson's disease), or frequent long-term use of antidepressants and other neurological drugs, (3) individuals with diseases affecting bone metabolism, such as liver and kidney disease, diabetes, bone tumors, or bone and joint diseases, (4) prolonged use of drugs impacting bone metabolism like calcium and hormones, (5) individuals lacking information on dietary intake, having missing or incomplete data, or refusing to participate. The investigation received approval from the Medical Research Ethics Committee of the Qingdao Center for Disease Control and Prevention (QDU-HEC-2023250), and all study participants provided informed consent. Participants could withdraw at any time.

**Figure 1:**
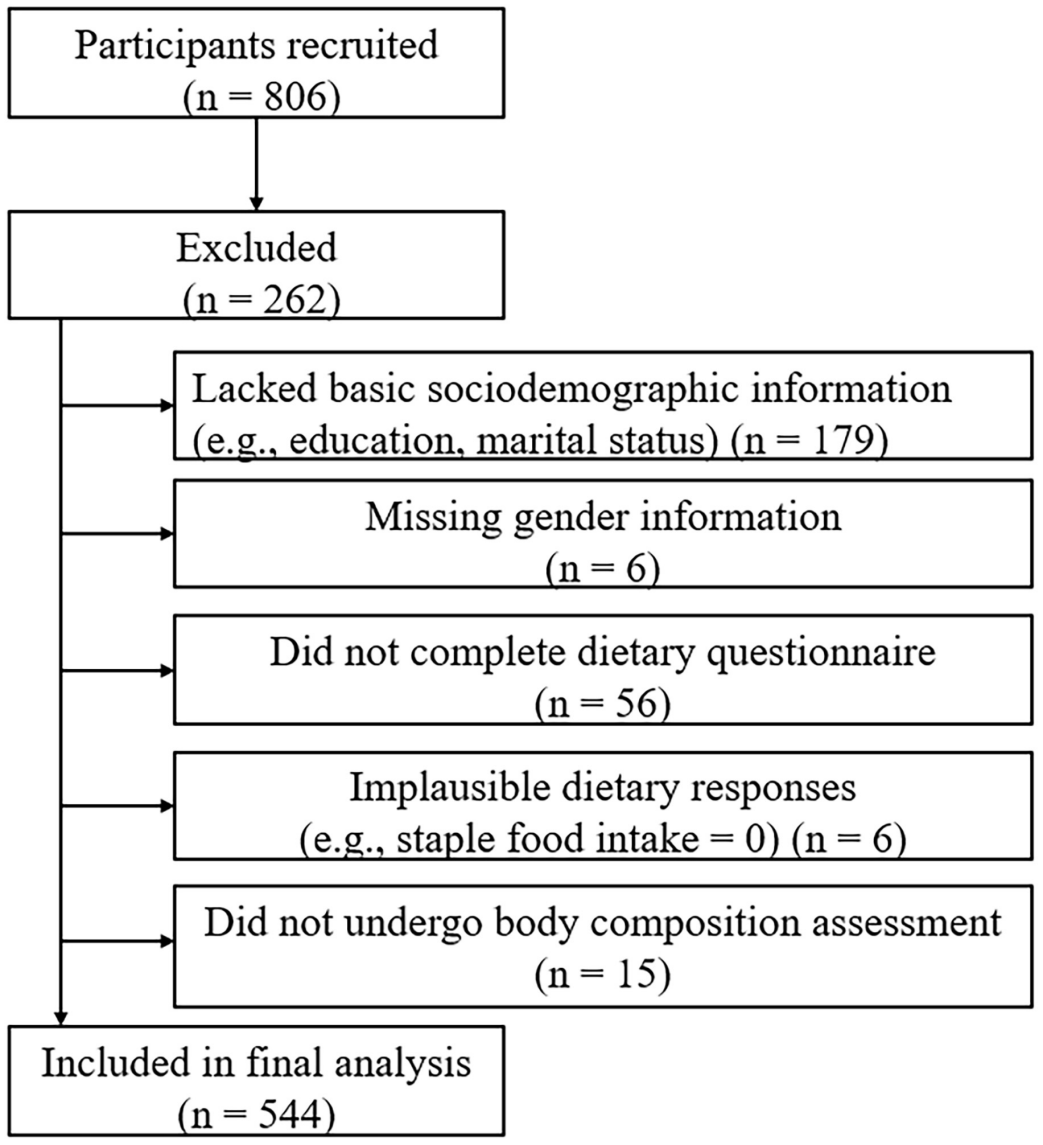
Flowchart of participant recruitment and inclusion.

A total of 806 participants were initially recruited. After excluding 179 participants who lacked basic sociodemographic information (e.g., education level, marital status), 6 participants without gender information, 56 participants who did not complete the dietary questionnaire, 6 participants with implausible dietary responses (e.g., staple food intake = 0), and 15 participants who did not undergo body composition assessment, a total of 544 participants were included in the final analysis (Figure 1).

### 2.2 Research content and methodology

#### 2.2.1 Participant characteristics

A comprehensive questionnaire was utilized to gather information regarding the overall profile of elderly individuals, including socio-demographic characteristics such as age, gender, education level, marital status, residence, history of chronic illness, disposable income, amount spent on food and drink, and time dedicated to various activities.

#### 2.2.2 Dietary information

The study used the Food Frequency Questionnaire (FFQ) to assess participants' dietary intake over the past 3 months, exploring the types, frequency, and quantities of foods consumed. Each participant received a food diary and a measurement sheet featuring life-size depictions of spoons, cups, and bottles. Participants who responded to < 10% of the FFQ data were deemed invalid and excluded from the study. Unreported food items were assumed not to be consumed during the recall period. FFQ responses were converted into daily equivalents and categorized based on the nutritional composition similarities of the food items. Based on the “Chinese Dietary Guidelines for Residents,” we comprehensively considered the nutritional characteristics of food and its prevalence in residents' daily diets, and selected the main food groups for inclusion in the analysis. In terms of food classification, we have adopted internationally recognized classification standards (Codex Alimentarius) that are in line with the dietary habits of Chinese residents, such as dividing livestock and poultry meat into red meat and white meat, etc., to ensure the scientific and reasonable nature of the classification. The 97 items were classified into 19 food groups (g/week), which included edible oils, salt, sugar, whole grains, pulses, vegetables, preserved products, fruits, dairy, white meat, red meat, offal, seafood, eggs, nuts, tea and coffee, fried and non-fried rice and noodles, and non-fried potatoes. The grouping of foods is detailed in Supplementary Table 1.

#### 2.2.3 Classification of meal patterns

Principal component analysis and maximum variance orthogonal rotation were used to extract the main dietary patterns, following these steps: firstly, the data samples underwent standardization. Given that the units of food intake measured during the survey were not uniform (g or ml), the intake of each food type was converted to grams per week before conducting data analysis. Next, the determination of the number of principal factors was based on whether the cumulative contribution or characteristic root exceeded one. Finally, a maximum variance orthogonal rotation of the initial factor loading matrix was executed, and factors with absolute values of factor loadings >0.40 were retained in the dietary pattern. These retained factors were named based on the shared characteristics of the included foods.

#### 2.2.4 Body composition analysis

Bioelectrical Impedance Analysis (BIA; DBA-510, Donghua Yuan Medical) was used to assess body composition. Participants were instructed to fast, empty their bowels, and avoid exercise before measurement. Trained technicians recorded values including total protein, muscle mass, fat-free mass, and skeletal muscle mass.

#### 2.2.5 Diagnostic criteria for osteoporosis

Bone density, measured using an ultrasound bone densitometer, followed the World Health Organization's diagnostic criteria. Normal bone mass was defined as T ≥ −1, reduced bone mass as −2.5 < T < −1, and osteoporosis as T ≤ −2.5. However, the use of ultrasonic bone density instruments instead of the gold standard DXA for diagnosing osteoporosis in this study has limitations. For example, ultrasonic bone density instruments are not very accurate in measuring BMD in elderly people with comorbidities. In addition, the physiological characteristics of the elderly population can also lead to inaccurate measurement results from ultrasonic bone density instruments.

#### 2.2.6 Quality control

The researcher enhanced compliance by elucidating the study's purpose and significance to participants. Face-to-face interviews were conducted between the researcher and participants for questionnaire completion. Researchers underwent standardized skills training before the survey to minimize measurement and survey bias. Physical examinations were performed by professionals using calibrated instruments. All data were double-entered, and consistency checks were conducted.

#### 2.2.7 Statistical analysis

Double data entry was performed using EpiData 3.1 software, and the results were analyzed using SPSS 26.0 statistical software and R version 4.4.1 (2024-06-14 ucrt). The study population was categorized into osteoporotic and non-osteoporotic groups based on T-score results. Continuous variables were expressed as mean and standard deviation (SD) if normally distributed, otherwise as median and interquartile range (IQR). Comparisons between osteoporosis and normal groups employed *t*-tests or Wilcoxon rank sum tests for continuous variables and the Pearson chi-square test or Fisher's exact test for categorical variables. Participants were categorized into quartiles based on their factor scores for each dietary pattern. Logistic regression explored the relationship between dietary patterns and osteoporosis, with the lowest quartile as the reference. Analysis of variance and rank sum test were used to analyze the relationship between dietary patterns and body composition. The researchers used the Spearman's rank correlation method in the R software to analyze the correlation between body composition and T-score. And R software was further used to draw the restricted cubic splines of high-protein dietary patterns with osteoporosis, body composition, and body composition with T-score. Given the significant association between age and osteoporosis, a sensitivity analysis was conducted by stratifying participants into two age groups (60–70 years and ≥70 years). Logistic regression and rank-sum tests assessed the associations between the high-protein dietary pattern, osteoporosis, and body composition.

## 3 Results

### 3.1 Basic information about the study population

Table 1 summarizes sample characteristics associated with various measures of osteoporosis. Significant differences in age and residence status were observed between individuals with osteoporosis and those with normal bone mass (*p* < 0.001). Younger age was correlated with a more favorable bone mass profile, especially among those living with a spouse. While the proportion of women with osteoporosis was higher, the difference was not statistically significant.

**Table 1:** General characteristics of the study subjects.

**Variables**	**Osteoporosis**		***p* ^†^**
	**Yes (*****n*** = **65)**	**No (*****n*** = **479)**	
**Sex^‡^**			0.515
Men	31 (47.7)	208 (43.4)	
Women	34 (52.3)	271 (56.6)	
Age^§^ (years)	73.88 ± 5.9	71.17 ± 5.714	<0.001^*^
**Education^‡^**			0.482
Illiteracy	29 (44.6)	202 (42.2)	
Primary school	28 (43.1)	189 (39.5)	
Secondary and above	8 (12.3)	88 (18.4)	
**Marital status^‡^**			0.405
Unmarried	0 (0.0)	5 (1.0)	
Married	48 (73.8)	385 (80.4)	
Widowed	17 (26.2)	88 (18.4)	
Divorced	0 (0.0)	1 (0.2)	
**Living condition^‡^**			0.001^*^
Live alone	6 (9.2)	83 (17.3)	
Spouse	41 (63.1)	340 (71.0)	
Children	16 (24.6)	54 (11.3)	
Else	2 (3.1)	2 (0.4)	
**Annual net income^‡^** **(yuan)**			0.377
<1,000	8 (12.3)	31 (6.5)	
1,001–3,000	12 (18.5)	98 (20.5)	
3,001–5,000	10 (15.4)	81 (16.9)	
5,001–8,000	12 (18.5)	68 (14.2)	
>8,001	23 (35.4)	201 (42.0)	
**Annual dietary expenditure (yuan)**			0.709
<1,000	15 (23.1)	95 (19.8)	
1,001–3,000	17 (26.2)	139 (29.0)	
3,001–5,000	14 (21.5)	89 (18.6)	
5,001–8,000	9 (13.8)	53 (11.1)	
>8,001	10 (15.4)	103 (21.5)	
**Smoke^‡^**			0.511
Yes	22 (33.8)	143 (29.9)	
No	43 (66.2)	336 (70.1)	
**Drink**			0.309
Yes	23 (35.4)	140 (29.2)	
No	42 (64.6)	339 (70.8)	
**Chronic disease**			0.322
Nothing	37 (56.9)	241 (50.3)	
1	17 (26.2)	152 (31.7)	
2	9 (13.8)	49 (10.2)	
≥3	2 (3.1)	37 (7.7)	
BMI^§^	24.202 ± 4.144	24.618 ± 3.608	0.443

† T-test for continuous variables and Chi-square test for categorical variables.

‡ n (%).

§ Mean ± SD.

* *p* < 0.05.

### 3.2 Meal pattern building

The dietary pattern was analyzed through principal component analysis, and its suitability test resulted in KMO = 0.617, which is >0.6, and Bartlett's spherical test yielded *P* < 0.001. According to the criteria, a total of four dietary patterns were finally extracted. They were named as follows: balanced Dietary Pattern (comprising vegetables, fruits, red meat, and non-fried rice and noodles) with 10.292% explained variance; high Protein Dietary Pattern (including legumes, white meat, seafood, and non-fried potatoes) with 9.362% explained variance; condiment Dietary Pattern (consisting of edible oil, salt, tea, and coffee) with 8.261% explained variance; snack Dietary Pattern (involving pickled products, eggs, nuts, and fried items) explaining 8.208% of the variance. The cumulative variance contribution of these patterns was 36.123%. Detailed results can be found in Tables 2, 3.

**Table 2:** Rotated factor loading matrix for DPs.

**Food items**	**Dietary patterns**			
	**Balanced DP**	**High protein DP**	**Condiment DP**	**Snack DP**
Cooking oil	0.173	−0.022	0.743^†^	−0.170
Salt	0.266	−0.248	0.728^†^	0.099
Sugar	−0.101	−0.019	0.057	0.325
Whole grain	−0.360	0.011	0.035	0.310
Beans	0.010	0.618^†^	0.022	0.021
Vegetables	0.586^†^	0.354	0.035	−0.052
Pickled products	0.047	0.229	−0.064	0.604^†^
Fruit	0.528^†^	0.146	0.106	0.166
Milk	−0.042	0.354	−0.119	−0.036
White meat	0.267	0.529^†^	−0.134	0.140
Red Flesh	0.554^†^	0.240	0.117	0.076
Viscera	−0.138	0.310	0.354	0.122
Seafood category	0.219	0.510^†^	0.149	0.123
Egg	0.398	−0.146	0.016	0.452
Nuts	0.370	−0.101	0.030	0.530^†^
Tea Coffee	−0.293	0.245	0.462	0.014
Deep-fried	−0.085	0.156	−0.069	0.584^†^
Non-fried rice and noodles	0.486	−0.052	−0.030	−0.198
Non-fried potatoes	0.136	0.445	0.227	0.022

DPs, Dietary patterns.

† Absolute values ≥0.4.

**Table 3:** Cumulative contribution of variance.

**Ingredient**	**Rotate square and load**		
	**Feature root**	**Variance contribution rate (%)**	**Cumulative variance contribution rate (%)**
Balanced dietary pattern	1.965	10.292	10.292
High protein dietary pattern	1.779	9.362	19.654
Condiment dietary pattern	1.57	8.261	27.915
Snack dietary pattern	1.559	8.208	36.123

### 3.3 Relationship between osteoporosis and dietary patterns

The odds ratio (OR) with a 95% confidence interval for the presence or absence of osteoporosis revealed a significant positive association with bone health at higher factor scores for the high protein dietary pattern [Q3: OR (95% CI) 0.435(0.196, 0.962); Q4: OR (95% CI) 0.435(0.196, 0.962)]. This positive association persisted after adjusting for gender [Q3: OR (95% CI) 0.423 (0.191, 0.939); Q4: OR (95% CI) 0.425 (0.192, 0.942)]. After further adjustments for potential confounders (age, residence status), the residential factor refers to living alone, having a spouse, living with children, living in a nursing home, and other situations. The high protein dietary pattern was found to be positively associated with bone health at the Q3 level [OR (95% CI) 0.435 (0.190, 0.997)], while no correlation was observed at the Q4 level. Detailed information is presented in Table 4. However, no relationship was observed between other dietary patterns and bone health. According to the sensitivity analysis by age groups, the association between high-protein dietary pattern and osteoporosis observed in the entire sample was not statistically significant in both subgroups (see Supplementary Tables 2, 4). The results of the RCS (restricted cubic spline) test for the high-protein dietary pattern and osteoporosis showed that the nonlinear test (with p indicating nonlinearity) had no statistical significance, suggesting that the observed association might be essentially linear (see Supplementary Figure 1).

**Table 4:** Logistic regression analysis and chi-square of dietary pattern and osteoporosis.

**Dietary patterns**	**Osteoporosisn (%)**		**Model I**	**Model II**	**Model III**		
	**Yes**	**No**	**OR (95% CI)**	**OR (95% CI)**	**OR (95% CI)**	**Card side**	***P***
**Balanced DP**						0.192	0.979
Q1	16 (24.6)	120 (25.1)	1	1	1		
Q2	17 (26.2)	119 (24.8)	1.071 (0.517, 2.219)	1.093 (0.526, 2.269)	0.983 (0.462, 2.094)		
Q3	15 (23.1)	121 (25.3)	0.930 (0.440, 1.965)	0.938 (0.444, 1.984)	0.939 (0.446, 1.979)		
Q4	17 (26.2)	119 (24.8)	1.071 (0.517, 2.219)	1.076 (0.519, 2.229)	0.855 (0.397, 1.839)		
**High protein DP**						11.235	0.011^*^
Q1	21 (32.3)	115 (24.0)	1	1	1		
Q2	24 (36.9)	112 (23.4)	1.173 (0.618, 2.227)	1.176 (0.619, 2.234)	1.223 (0.628, 2.379)		
Q3	10 (15.4)	126 (26.3)	**0.435 (0.196, 0.962)**	**0.423 (0.191, 0.939)**	**0.435 (0.190, 0.997)**		
Q4	10 (15.4)	126 (26.3)	**0.435 (0.196, 0.962)**	**0.425 (0.192, 0.942)**	0.462 (0.204, 1.045)		
**Condiment DP**						3.171	0.366
Q1	19 (29.2)	117 (24.4)	1	1	1		
Q2	20 (30.8)	116 (24.2)	1.062 (0.539, 2.092)	1.050 (0.532, 2.071)	1.075 (0.525, 2.199)		
Q3	14 (21.5)	122 (25.5)	0.707 (0.339, 1.474)	0.694 (0.332, 1.451)	0.829 (0.385, 1.783)		
Q4	12 (18.5)	124 (25.9)	0.596 (0.277, 1.281)	0.580 (0.269, 1.251)	0.616 (0.270, 1.403)		
**Snack DP**						1.61	0.657
Q1	19 (29.2)	117 (24.4)	1	1	1		
Q2	18 (27.7)	118 (24.6)	0.939 (0.469, 1.879)	0.941 (0.470, 1.883)	1.097 (0.535, 2.251)		
Q3	13 (20.0)	123 (25.7)	0.651 (0.308, 1.377)	0.646 (0.305, 1.368)	0.673 (0.308, 1.472)		
Q4	15 (23.1)	121 (25.3)	0.763 (0.370, 1.573)	0.751 (0.364, 1.551)	0.846 (0.398, 1.798)		

Model I, No adjustment for confounding factors; Model II, Adjusted for gender; Model III, Adjusted for age and residence confounding factors.

* *p* < 0.05. Bold values indicate 95% CIs excluding the null value (1.0), representing statistically significant associations at P < 0.05.

### 3.4 Relationship between dietary patterns and body composition

To further explore the relationship between the high-protein dietary pattern and osteoporosis, we examined the differences in body components at different factor score levels of the high-protein dietary pattern. Analysis of variance and rank sum test were used to analyze the relationship between dietary patterns and body composition. Significant differences (*P* < 0.05) were observed in protein, muscle, inorganic salt, defatted body weight, skeletal muscle, left hand muscle mass, right hand muscle mass, and trunk muscle mass among groups at different score levels. No significant differences were found in the visceral fat area, hip circumference, waist-to-hip ratio, left leg muscle mass, right leg muscle mass, body fat, body mass index, body fat percentage, body obesity percentage, waist circumference, and total score. The median of each body component increased with the level of factor scores, reaching its highest at the Q3 level and decreasing at the Q4 level. Detailed results are presented in Tables 5, 6. The sensitivity analysis stratified by age groups revealed that the association between high-protein dietary patterns and body composition, as observed in the entire sample, was not statistically significant in either subgroup. This finding, along with the results of the aforementioned sensitivity analysis of high-protein dietary patterns and osteoporosis, may be attributed to reduced sample size and statistical power. Notably, the direction of the association remained consistent across groups, supporting the robustness of the overall finding (see Supplementary Tables 3, 5). The RCS test results of high-protein diet patterns and body composition show that the nonlinear test (p indicates nonlinearity) has no statistical significance, suggesting that the observed correlation may be essentially linear (see Supplementary Figure 3).

**Table 5:** The relationship between high protein dietary pattern and body composition P50 (P25, P75).

**Body composition**	**Q1 (*n* = 136)**	**Q2 (*n* = 136)**	**Q3 (*n* = 136)**	**Q4 (*n* = 136)**	**H**	***P***
Intracellular fluid	18.4 (16.4, 20.95)	18.25 (15.9, 20.45)	19.4 (17.05, 22.8)	18.8 (16.2, 22.15)	9.184	0.027^*^
Extracellular fluid	11.8 (10.7, 13.4)	11.7 (10.05, 13.2)	12.3 (10.8, 14.45)	12.1 (10.5, 14.1)	8.995	0.029^*^
Total water	30.15 (27.2, 34.25)	29.75 (26, 33.65)	31.85 (27.85, 37.25)	30.95 (26.8, 36.2)	9.099	0.028^*^
Protein	7.95 (7.1, 9.1)	7.9 (6.9, 8.8)	8.4 (7.35, 9.85)	8.1 (7.0, 9.55)	9.194	0.027^*^
Muscle	38.1 (34.3, 43.3)	37.65 (32.95, 42.50)	40.25 (35.25, 47.15)	39.15 (33.8, 45.8)	9.062	0.028^*^
Inorganic salt	3.105 (2.79, 3.495)	3.075 (2.655, 3.49)	3.25 (2.84, 3.76)	3.195 (2.79, 3.68)	9.187	0.027^*^
FFM	41.15 (37.15, 46.8)	40.65 (35.55, 45.95)	43.45 (38.05, 50.9)	42.25 (36.6, 49.5)	9.095	0.028^*^
Visceral fat area	132.55 (122.45, 147.55)	135.05 (121.25, 150.5)	134.05 (122.65, 152.5)	132.6 (119.6, 147.7)	1.174	0.759
Body fat rate	28.45 (24.4, 34.9)	30 (23.6, 35.3)	29.7 (22.35, 33.15)	27.65 (20.95, 34.15)	2.891	0.409
Waist-to-hip ratio	0.97 (0.950, 0.990)	0.97 (0.950, 0.990)	0.965 (0.950, 0.990)	0.97 (0.940, 0.985)	1.902	0.593
Skeletal muscle	22 (19.4, 25.35)	21.8 (18.75, 24.7)	23.3 (20.25, 27.75)	22.5 (19.1, 26.9)	9.187	0.027^*^
Left hand muscle mass	2.22 (1.92, 2.61)	2.21 (1.815, 2.60)	2.375 (2.03, 2.865)	2.285 (1.89, 2.845)	8.576	0.035^*^
Right hand muscle mass	2.23 (1.90, 2.59)	2.21 (1.825, 2.60)	2.37 (2.025, 2.89)	2.305 (1.89, 2.86)	8.648	0.034^*^
Trunk muscle mass	19.04 (17.27, 21.56)	19.09 (16.44, 21.395)	19.89 (17.76, 23.08)	19.615 (16.85, 22.78)	8.801	0.032^*^
Left leg muscle mass	6.105 (5.195, 7.065)	5.985 (4.96, 7.105)	6.45 (5.395, 7.61)	6.14 (5.205, 7.525)	7.157	0.067
Right leg muscle mass	6.1 (5.185, 7.19)	5.95 (5.00, 7.07)	6.41 (5.385, 7.857)	6.07 (5.21, 7.56)	7.059	0.07

* *p* < 0.05.

**Table 6:** The relationship between high protein dietary pattern and body composition (Mean ± standard deviation).

**Body composition**	**Q1 (*n* = 136)**	**Q2 (*n* = 136)**	**Q3 (*n* = 136)**	**Q4 (*n* = 136)**	**F**	***P***
Body fat	17.65 ± 6.6405	17.485 ± 6.5233	18.161 ± 7.0294	16.846 ± 7.0419	0.861	0.461
Body mass index	24.4 ± 3.5568	24.36 ± 3.6949	24.815 ± 3.7544	24.021 ± 3.6209	1.076	0.359
Hip circumference	90.629 ± 6.1268	90.243 ± 5.7131	92.075 ± 6.6743	90.212 ± 6.7156	2.621	0.050
Body obesity rate	114.077 ± 17.1093	113.924 ± 17.9862	115.417 ± 17.6258	111.86 ± 17.4664	0.952	0.415
Waist circumference	87.714 ± 7.3824	87.51 ± 7.1600	89.254 ± 8.1320	87.463 ± 8.1185	1.663	0.174
The total score	71.068 ± 4.4701	71.088 ± 4.0749	71.26 ± 4.2788	71.604 ± 4.5950	0.441	0.724

Method: Analysis of variance. F: Variance test.

### 3.5 Relationship between body composition and T-value

**Figure 2:**
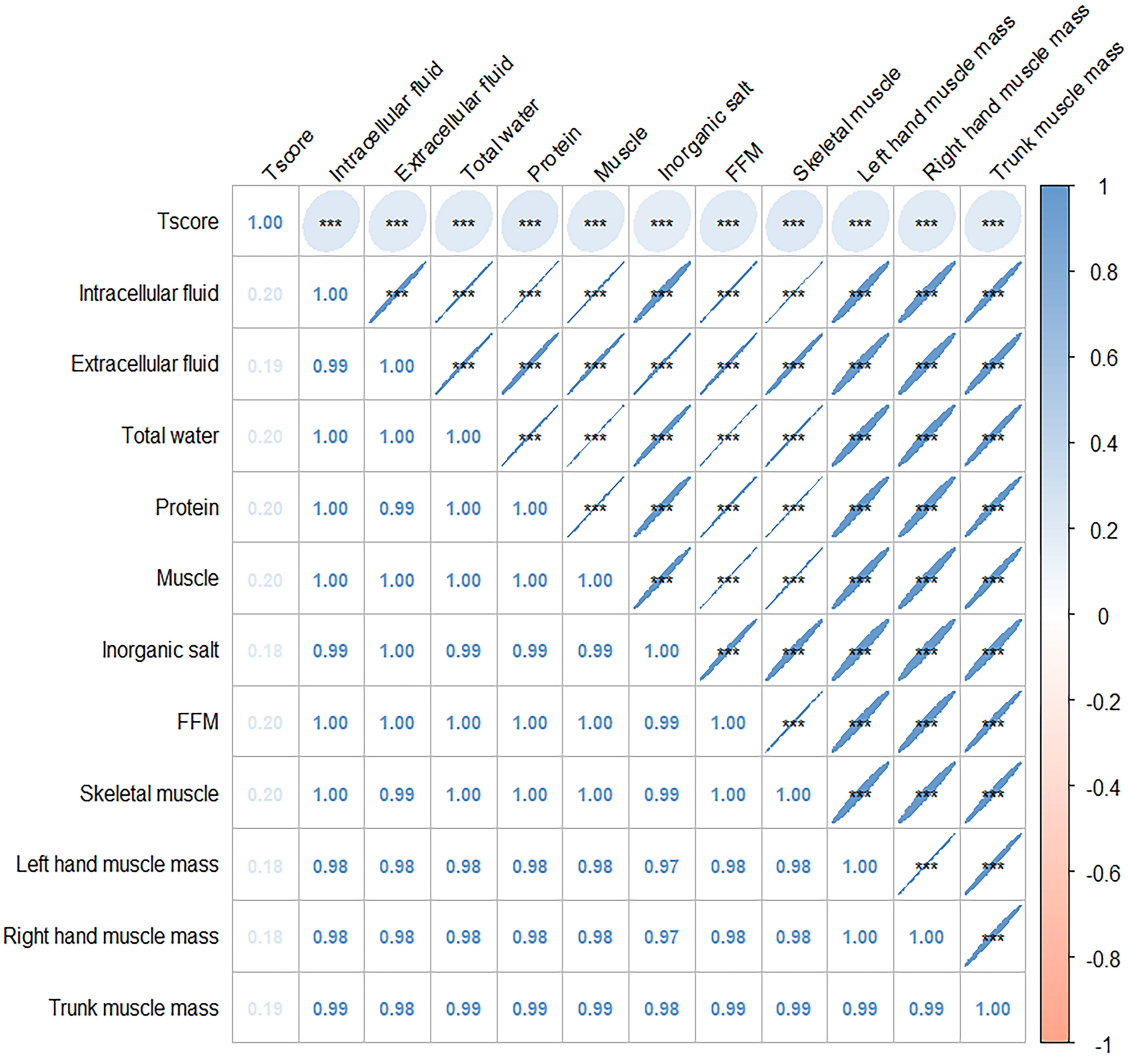
Correlation plot of Spearman correlation coefficients between body composition and T score. Annotation: The numbers indicate the Spearman's correlation coefficients. Significant values are shown as ***P < 0.01.

**Figure 3:**
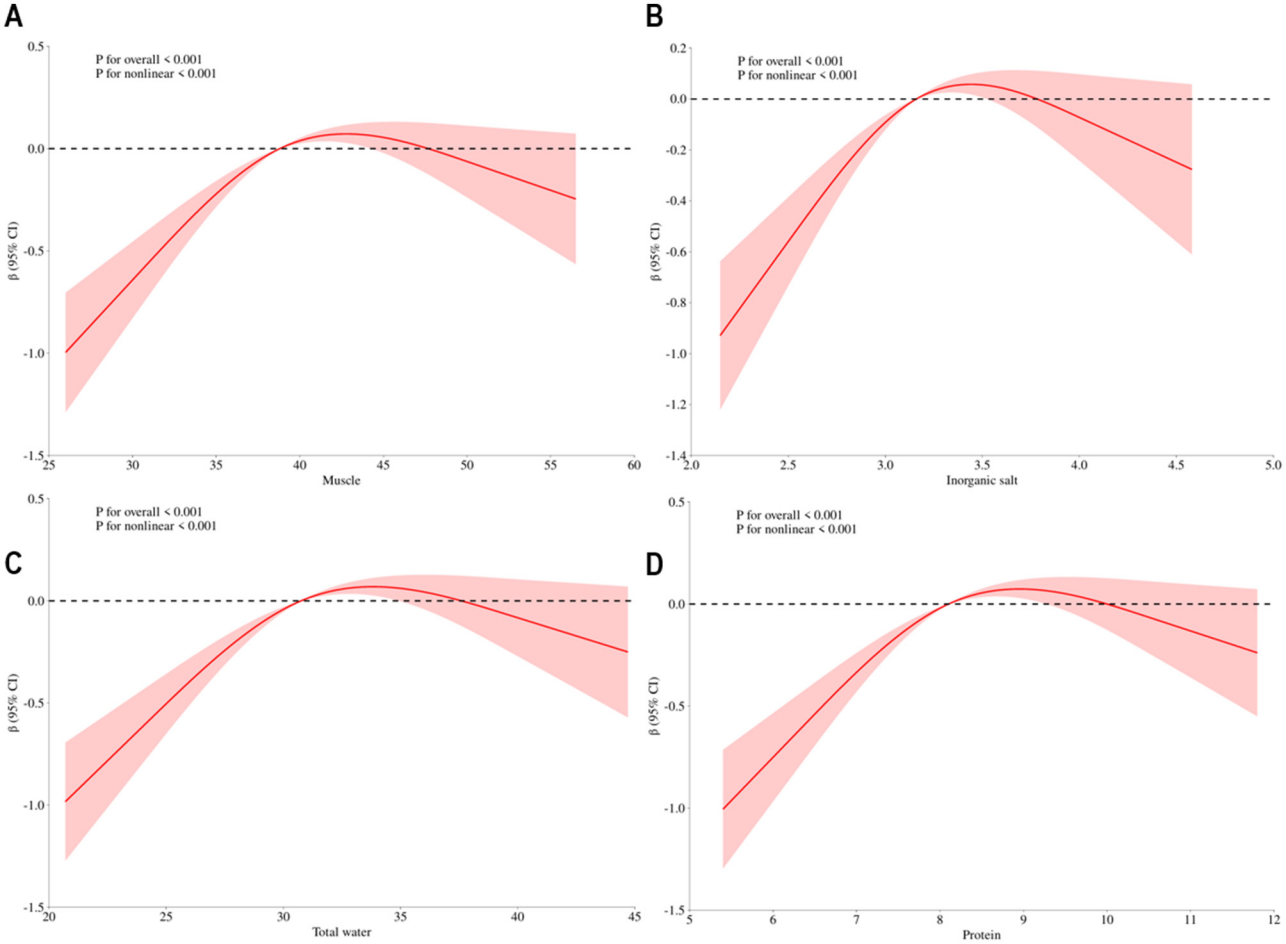
Dose-response association between body composition and t-score. Annotation: **(A)** Dose-response association between muscle and t-score, **(B)** Dose-response association between inorganic salt and t-score, **(C)** Dose-response association between total water and t-score, **(D)** Dose-response association between protein and t-score. Red solid line represents estimates off odds ratio.

The connection between body composition and osteoporosis was explored through Spearman's rank correlation analysis of body composition and T-score. The analysis revealed a correlation between components that exhibited significant differences in various factor score levels (protein, muscle, inorganic salt, defatted body weight, skeletal muscle, left hand muscle mass, right hand muscle mass, trunk muscle mass, and right leg muscle mass) and T-score. Detailed results are presented in Figure 2. The results of the restricted cubic spline (RCS) test for body composition (protein, muscle, inorganic salts, and total body water) and T-score showed that all nonlinear tests were statistically significant (*p* < 0.001). The inflection points for protein, muscle, total water, and inorganic salts are 8.1, −38.8, 30.75, and 3.16, respectively (see Figure 3).

## 4 Discussion

Osteoporosis, a systemic skeletal disease characterized by a decrease in bone mass and the destruction of bone microstructure, has become a global public health problem. In this study, the prevalence of osteoporosis among the elderly aged 60 and above was 11.95%. This figure is comparable to the prevalence of 13.4% among people aged 50 and above reported by Zeng et al. based on a national multi-center study (38). It is worth noting that a meta-analysis from China showed that the prevalence of osteoporosis among the elderly reached 18.9% (39). Previous studies have indicated that the prevalence of osteoporosis is higher among Asian elderly people (40). In a systematic review that included multiple studies, it was found that the prevalence rates of osteoporosis in Asia, Europe, and the United States were 24.3%, 16.7%, and 11.5%, respectively (41). The differences in prevalence rates among different study cohorts suggest that dietary patterns may be an important influencing factor. The characteristic of high intake of high-quality proteins such as legumes in the population of this study may be an important reason why the prevalence rate in this population is lower than the domestic average level. The Mediterranean diet pattern, which is popular in the United States, is beneficial to bone health (42). The Mediterranean diet is characterized by being rich in fruits, vegetables, whole grains, and fish, and it advocates the intake of nutrients with anti-inflammatory effects, such as dietary fiber, omega-3 fatty acids, monounsaturated fatty acids (MUFA), and polyphenols in olive oil (OO). These nutrients may have a positive impact on reducing the process of bone resorption and muscle wasting (43, 44). However, the deficiency of vitamins and dietary deficiencies such as calcium among the Asian population may contribute to the relatively high prevalence of osteoporosis among Asians (45).

While nutrition and bone health have long been focal points in public health, studies exploring the relationship between dietary patterns and osteoporosis in the elderly remain limited, and research focusing on rural regions of China is notably scarce. Our study analyzed the dietary patterns of elderly residents in a rural area of Qingdao, China. We found that a high-protein dietary pattern is significantly associated with a reduced risk of osteoporosis and demonstrates a positive correlation with T-scores, a key indicator of bone mineral density. These findings are largely consistent with previous research. Several studies have reported a positive correlation between bone mineral density (BMD) and protein intake (46, 47). A cross-sectional study found that low protein intake may increase the risk of osteoporosis in adults over 45 years of age (48), while other Animal studies have also linked low protein consumption to decreased bone mass (49, 50). Moreover, a systematic review found that a relatively high intake of protein from low-fat or skimmed dairy products in the population is associated with an improvement in bone health status (51). Dietary protein intake was reported to be associated with bone density in in a prospective cohort study of 2,160 people (52). In contrast, a meta-analysis of the U.S. population showed that a higher protein intake had no statistically significant beneficial effect on bone mineral density (BMD) (53). The BMD of the lumbar spine was only slightly improved, and there was no impact on the BMD of the total hip, the BMD of the femoral neck, or bone markers (54).

Dietary protein intake can have a direct positive influence on bone structure and function. A protein-rich diet contributes to sustaining the functionality of the skeletal system and body muscles, reducing the risk of complications associated with osteoporosis (55). There are some mechanisms as follows: firstly, the extracellular matrix of bone is mainly composed of collagen, and its major component, type I collagen, plays an important role in maintaining the structural integrity of the collagen network in bone. Protein is involved in collagen synthesis (56–58). Importantly, collagen determines the amount of mineral deposition. Thus, the capacity of bone to resist mechanical forces and fractures depends not only on the quantity of bone tissue (mineralization) but also its quality (organization of the collagen framework) (59, 60). Secondly, many hormones, kinases, and growth factors involved in bone formation, such as TGF-β and BMP, are composed of proteins (61, 62). For instance, BMP (bone morphogenetic protein) can induce bone and cartilage formation. Some cytokines, such as RUNX2 and SOX9, function downstream of BMP, which have a positive effect on bone health (63). Studies have found that IGF-1, which is widely concerned at present, is related to protein intake (64). For instance, IGF-1 (insulin-like growth factor-1) plays a crucial role in bone remodeling, influencing osteoblast differentiation, proliferation, and the function of osteoclasts, thereby affecting bone mineralization (65). Thirdly, the study found that the interaction between protein metabolism and gut microbiota has a special regulatory mechanism for bone health. The gut microbiota generates short-chain fatty acids (such as acetic acid and propionic acid) through the metabolism of proteins, regulating the process of bone remodeling. There is an association between changes in the microbiota structure (such as an increase in Bacteroidetes and a decrease in Firmicutes) and the improvement of bone metabolism (66–70). These mechanisms echo the Spearman results of the analysis on body composition and t-score, indicating that components with significant differences in different factor score levels of high-protein dietary patterns are correlated with T-scores. Additionally, the RCS analysis of body composition and T-score reveals that body composition indices such as protein, muscle, total water, and inorganic salts all show a positive correlation with T-score before reaching a certain threshold, with no discernible trend beyond this threshold. Last, further insights obtained through body composition testing in our study unveiled that in a high-protein dietary pattern, key body composition indicators such as protein content, skeletal muscle, and muscle mass demonstrated an increasing trend with rising intake. These changes in body composition mainly reflect changes in protein in the body. The reason may be that high protein intake can stimulate the availability of amino acids in the whole body and thus increase protein synthesis so that the digested and absorbed amino acids stimulate muscle protein formation (71). In summary, it can be seen that a high-protein diet has certain benefits for bones.

Dietary protein intake not only affects bone structure and function but also plays a crucial role in calcium balance. Notably, studies have shown that excessive protein consumption increases urinary calcium excretion, which may negatively affect bone integrity. A series of carefully controlled metabolic balance studies have shown that a high-protein diet is harmful to bone health because the metabolic acidity from protein catabolism can lead to an increase in urinary calcium excretion (72, 73). In addition, many studies have indicated that for the elderly population, increasing the dietary calcium intake level may not necessarily result in a clinically significant reduction in the risk of fractures, nor is it easy to achieve a sustained improvement in bone density (74, 75). Our findings are consistent with these studies, suggesting that a high-protein dietary pattern at the Q3 level may represent an optimal intake range for supporting bone health. The lack of additional benefit and potential adverse effects at the Q4 level supports the hypothesis of a non-linear dose-response relationship, where excessive protein intake may disrupt calcium balance and counteract bone protection.

Notably, the dietary patterns of rural elderly individuals exhibit unique characteristics. A study on the elderly in rural areas of Northeast China shows that due to the relatively limited food resources in rural areas, the dietary structure of the elderly is relatively simple, and they mostly take grains and vegetables grown at home as the staple food (76). Furthermore, the consumption proportions of fruits, dairy products, and vegetables have reached the recommended value rather low. Existing research data indicate that the intake of the above-mentioned food categories by Chinese residents is seriously insufficient (77, 78). In contrast, the dietary patterns of the elderly in cities are more diverse. A survey targeting the elderly population in cities shows that the abundant food supply in cities makes the elderly have more diverse food choices (79–81). In terms of protein intake, in addition to obtaining protein from traditional sources such as meat and eggs, urban elderly people also choose various sources such as soy products and dairy products, with a relatively high proportion of high-quality protein (82). In terms of calcium intake, urban elderly people consume dairy products such as milk, cheese and yogurt, as well as calcium-rich foods like soy products and seafood. Their intake content is relatively higher than that of rural elderly people, which is helpful for maintaining bone health (82). Numerous studies have shown that a healthy dietary pattern is beneficial to bone health. A healthy dietary pattern rich in vegetables, fruits, and high protein can help reduce the risk of fractures (83–86). Although rural elderly people have an adequate intake of vegetables, they do not consume enough high-quality protein, while some urban people have their bone health affected by poor dietary patterns. Therefore, it is necessary to further study the correlation between dietary patterns and bone health in rural populations.

This study focuses on rural elderly individuals, a population often overlooked in previous studies, particularly within China. Given the rural population's unique economic conditions, dietary habits, and limited nutritional knowledge, exploring their dietary patterns and bone health provides valuable insights into potential urban-rural differences. The use of quantitative ultrasound to obtain T-scores enhances the reliability of osteoporosis diagnosis. Moreover, applying dietary pattern analysis moves beyond the limitations of single-nutrient studies, reflecting overall dietary behaviors more realistically and improving the practical relevance of our findings. However, the cross-sectional design prevents causal inference, and unmeasured confounders such as vitamin D levels and calcium intake may affect the results. Additionally, the study's geographic limitation to Jiaozhou, Qingdao, may restrict generalizability to other regions. Further longitudinal and multicenter studies are needed to validate these findings.

## 5 Conclusion

Principal component analysis identified four distinct dietary patterns. Among these, one pattern characterized by elevated consumption of legumes, white meat, seafood, and non-fried potatoes was significantly associated with improved bone health. However, additional large-scale, population-based studies are required to confirm these associations and evaluate their generalizability.

## Data Availability

The raw data supporting the conclusions of this article will be made available by the authors, without undue reservation.
