# Supplementary material for: Study on the correlation between dietary patterns and bone health among rural elderly people in Qingdao
Source: Front Nutr. 2025 Sep 5;12:1613065. doi: 10.3389/fnut.2025.1613065 (PMC12446372; doi:10.3389/fnut.2025.1613065)
Supplement: Supplementary file 1 [file Table_1.docx]

**Supplementary Material**

**Study on the Correlation between Dietary Patterns and Bone Loss among Rural Elderly People in Qingdao**

Haoran Chang^1^, Wenchao Han^2^, Jiafei Li^1^, Dazhi Jiao^1^, Fangyu Liu^1^, Tianlin Gao^1^, Wenjing Zhu^3^, Jianbao Gong^4^*

^1^School of Public Health, Qingdao University, Qingdao, China

^2^Department of Pediatrics, Qingdao Hospital, University of Health and Rehabilitation Sciences(Qingdao Municipal Hospital), Qingdao, China

^3^ Department of Medical Research, Qingdao Hospital, University of Health and Rehabilitation Sciences(Qingdao Municipal Hospital), Qingdao, China

^4^***** Department of orthopedics surgery， Qingdao Hospital， University of Health and Rehabilitation Sciences(Qingdao Municipal Hospital), Qingdao, China

**Contents**

Supplementary table 1. Food groupings

| Food types | Specific food |
| --- | --- |
| Non-fried rice and noodle dishes | Rice，non-fried pasta |
| Deep Fried | Deep-fried pasta, instant noodles, salted soda crackers, salted mooncakes, sweet biscuits, buns, cakes, dim sum and mooncakes, fried crisps, Chips, other fried snacks |
| Whole grains | Whole grain |
| Vegetables | Fresh bean vegetables, tomatoes, peppers, carrots, melon vegetables, green leafy vegetables, cabbage and other leafy vegetables, cruciferous vegetables, other fresh or frozen vegetables, onions and garlic, mushrooms and algae, dried vegetables |
| Fruits | Orange fruit, watermelon, melon, other melon fruit, all other fresh frozen fruit, all other dried fruit other than dried fruit, 100% fruit juice |
| Red meats | Lean pork, fatty pork, beef, lamb, mutton, other non-processed meat |
| Non-fried potatoes | Sweet potato, potato, taro |
| Cooking oil | All cooking oils, lard |
| Salt | Soy sauce, monosodium glutamate, chicken essence, yellow sauce |
| Sugar | Chocolate, artificial sugary drinks including carbonated drinks and commercial teas, sugary drinks |
| Tea, coffee | Tea, coffee |
| Seafood | Fish, shrimp, crab or other seafood |
| White meat | Chicken, duck, goose, pigeon, quail |
| Pickled products | Pickled vegetables, fermented foods, dried fruit including canned or candied fruit, processed meat products, puffed eggs |
| Nuts | Nuts |
| Milk | Whole liquid milk, low-fat liquid milk, skimmed liquid milk, whole milk powder, low-fat milk powder, skimmed milk powder, yoghurt, cheese, ice cream |
| Eggs | Eggs |
| Beans | Other miscellaneous grains, dried soybeans, soy milk, soy flour, tofu, bean curd skin, other beans |
| Offal | Poultry and livestock offal |

Supplementary table 2. Logistic Regression and Chi-Square Analysis Stratified by Age Groups (60–70 Years)

| Dietary patterns | Osteoporosis | | Model I | Model II | Model III | Card side | P |
| --- | --- | --- | --- | --- | --- | --- | --- |
|  | Yes | No |  |  |  |  |  |
| High protein DP |  |  |  |  |  | 4.466 | 0.215 |
| Q1 | 5(31.25) | 115(24.0) | 1 | 1 | 1 |  |  |
| Q2 | 6(37.50) | 112(23.4) | 1.159(0.335,4.013) | 1.144(0.324,4.036) | 1.203(0.333,4.347) |  |  |
| Q3 | 1(6.25) | 126(26.3) | 0.184(0.021,1.620) | 0.152(0.017,1.366) | 0.101(0.008,1.221) |  |  |
| Q4 | 4(25.00) | 126(26.3) | 0.772(0.197,3.025) | 0.662(0.165,2.650) | 0.521(0.120,2.265) |  |  |

Model I： No adjustment for confounding factors; Model II： Adjusted for gender; Model III： Adjusted for age and residence confounding factors. * p<0.05

Supplementary table 3. The Relationship Between High Protein Dietary Pattern and Body Composition in Individuals Aged 60–70: P50 (P25, P75)

| Body composition | Q1（n=61) | Q2（n=64) | Q3（n=62) | Q4（n=62) | H | P |
| --- | --- | --- | --- | --- | --- | --- |
| Intracellular fluid | 19.200(17,500,21.850) | 19.25(18.025,21.875) | 20.450(18.375,23.850) | 20.450(17.475,22.975) | 5.001 | 0.172 |
| Total water | 31.300(28.600,35.650) | 31.550(29.525,35.475) | 33.350(29.925,38.900) | 33.500(28.600,37.600) | 4.821 | 0.185 |
| Protein | 8.300(7.550,9.450) | 8.350(7.800,9.400) | 8.850(7.900,10.325) | 8.850(7.575,9.925) | 4.992 | 0.172 |
| Inorganic salt | 3.200(2.895,3.615) | 3.230(2.993,3.565) | 3.370(3.050,3.855) | 3.390(2.868,3.735) | 4.595 | 0.204 |
| FFM | 42.800(39.000,48.700) | 43.050(40.400,48.475) | 45.600(40.900,53.125) | 45.800(39.075,51.350) | 4.857 | 0.183 |
| Body fat rate | 119(104.350,130.550) | 116.700(106.825,128.275) | 117.950(108.775,127.300) | 110.350(100.800,127.875) | 4.291 | 0.232 |
| Visceral fat area | 131.100(118.550,143.000) | 131.300(117.975,146.300) | 132.600(119.350,145.375) | 123.400(113.175,143.350) | 3.107 | 0.375 |
| Waist-to-hip ratio | 0.9600(0.9400,0.9800) | 0.9650(0.9500,0.9900) | 0.9600(0.9500,0.9825) | 0.9500(0.9300,0.9700) | 9.090 | 0.028 |
| Skeletal muscle | 23.100（20.850,26.550） | 23.100（21.525,26.550） | 24.650（21.975,29.150） | 24.700（20.775,28.000） | 4.993 | 0.172 |
| Right hand muscle mass | 2.400(2.035,2.760) | 2.425(2.165,2.725) | 2.575(2.138,3.013) | 2.510(1.080,2.923) | 4.510 | 0.211 |
| Left leg muscle mass | 6.270(5.415,7.270) | 6.325(5.645,7.395) | 6.700(5.730,8.080) | 6.780(5.500,7.743) | 3.345 | 0.341 |
| Right leg muscle mass | 6.280(5.490,7.320) | 6.400(5.668,5.540) | 6.660(5.808,8.133) | 6.865(5.475,7.795) | 4.008 | 0.261 |

Method ：Rank sum test. H: Kruskal-Wallis.

Supplementary table 4. Logistic Regression and Chi-Square Analysis Stratified by Age Groups (≥70 Years)

| Dietary patterns | Osteoporosis | | Model I | Model II | Model III | Card side | P |
| --- | --- | --- | --- | --- | --- | --- | --- |
|  | Yes | No |  |  |  |  |  |
| High protein DP |  |  |  |  |  | 1.492 | 0.684 |
| Q1 | 5(31.25) | 56（24.03） | 1 | 1 | 1 |  |  |
| Q2 | 4（25.00） | 46（19.74） | 0.974(0.247,3.833) | 0.925(0.231,3.709) | 1.082(0.264,0.433) |  |  |
| Q3 | 3（18.77） | 75（32.19） | 0.448(0.103,1.954) | 0.390(0.088,1.731) | 0.331(0,068,1.607) |  |  |
| Q4 | 4（25.00） | 56（24.04） | 0.800(0.204,3.136) | 0.705(0.176,2.817) | 0.568(0.848,1.643) |  |  |

Model I： No adjustment for confounding factors; Model II： Adjusted for gender; Model III： Adjusted for age and residence confounding factors. * p<0.05

Supplementary table 5. The Relationship Between High Protein Dietary Pattern and Body Composition in Individuals Aged ≥70 Years: P50 (P25, P75)

| Body composition | Q1（n=61) | Q2（n=50) | Q3（n=78) | Q4（n=60) | H | P |
| --- | --- | --- | --- | --- | --- | --- |
| Intracellular fluid | 19.200(17.700,21.800) | 19.350(18.100,21.800) | 20.250(18.300,23.800) | 20.300(17.450,22.450) | 4.939 | 0.176 |
| Extracellular fluid | 12.200(11.100,13.700) | 12.350(11.400,13.600) | 12.850（11.600,14.800） | 13.000(11.050,14.150) | 5.025 | 0.170 |
| Total water | 31.300(28.900,35.500) | 31.700(29.600,35.400) | 33.150(29.700,38.900) | 33.350(28.600,36.500) | 4.844 | 0.184 |
| Protein | 8.300(7.600,9.400) | 8.400(7.800,9.400) | 8.750(7.900,10.300) | 8.750（7.550,9.700） | 5.008 | 0.171 |
| Muscle | 39.600（36.500,45.000） | 40.100（37.400,44.900） | 41.900（37.500,49.300） | 42.100（36.100,46.200） | 4.868 | 0.182 |
| Inorganic salt | 3.200（2.900,3.600） | 2.230（3.000,3.550） | 3.370(3.050,3.840) | 2.865(3.365,3.680) | 5.135 | 0.162 |
| FFM | 42.800(39.400,48.500) | 43.350（40.400,48.400） | 45.350（40.600,53.100） | 45.550（39.050,49.900） | 4.893 | 0.180 |
| Visceral fat area | 131.100（119.000,142.600） | 131.300(117.500,143.900) | 132.600(119.900,146.500) | 123.150(113.150,142.750) | 3.949 | 0.267 |
| Waist-to-hip ratio | 0.960(0.940,0.980) | 0.965(0.950,0.990) | 0.960(0.950,0.990) | 0.950(0.930,0.970) | 8.390 | 0.039 |
| Skeletal muscle | 23.100(21.100,26.500) | 23.250(21.600,26.400) | 24.400(12.900,29.100) | 24.500(20.750,27.3000 | 4.932 | 0.177 |
| Left leg muscle mass | 6.270(5.450,7.250) | 6.395(5.790,7.380) | 6.665(5.720,8.050) | 6.720(5.480,7.715) | 2.437 | 0.487 |
| Right leg muscle mass | 6.280(5.490,7.300) | 6.485(5.900,7.450) | 6.640(5.690,8.100) | 6.810(5.460,7.705) | 2.864 | 0.413 |

Method ：Rank sum test. H: Kruskal-Wallis.


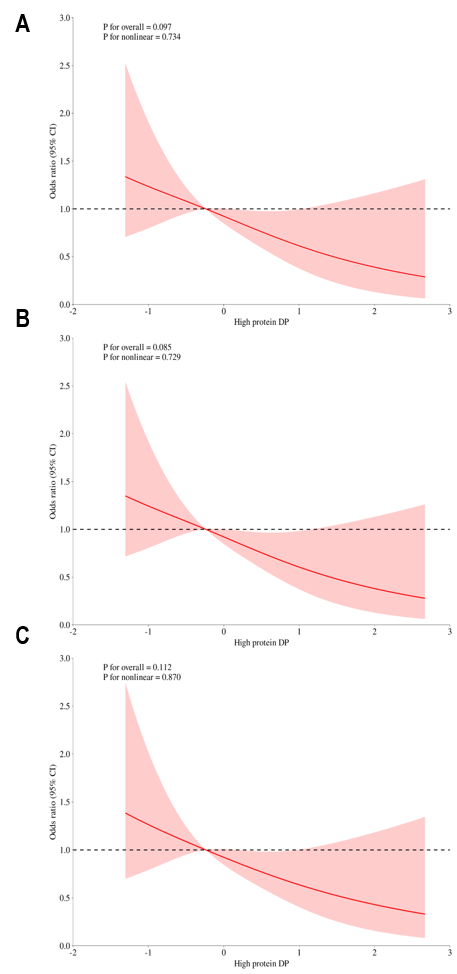


**Supplementary Figure 1.** Dose-response association between High protein DP and Osteoporosis. (A) No adjustment for confounding factors. (B)Adjusted for gender. (C)Adjusted for age and residence confounding. Red solid line represents estimates off odds ratio.


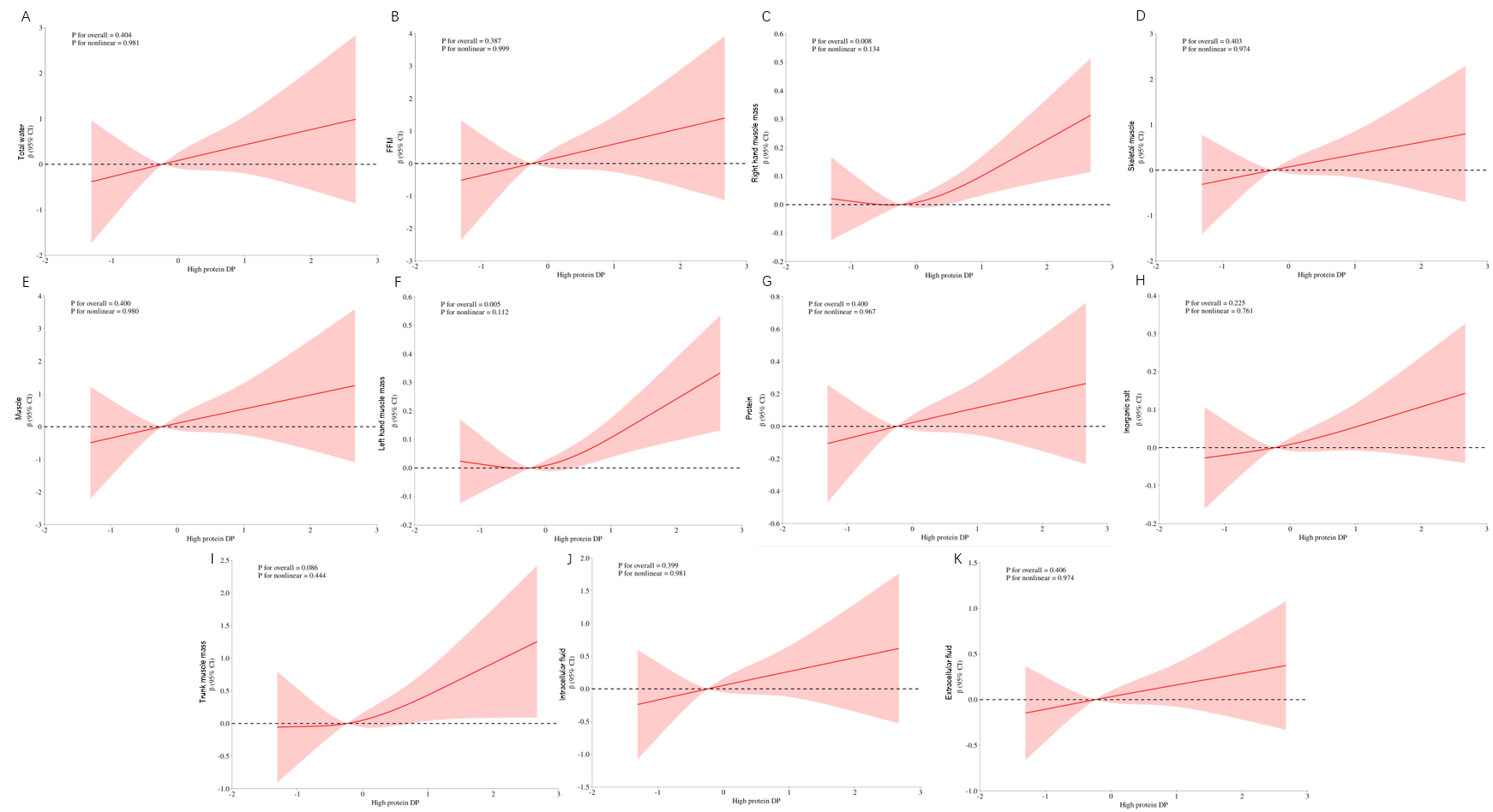


**Supplementary Figure 2.** Dose-response association between High protein DP and body composition. Red solid line represents estimates off odds ratio. FFM:fat free mass.
